# Food Knowledge, Habits, Practices, and Addiction Among Adolescents: A Cross-Sectional Investigation

**DOI:** 10.7759/cureus.47175

**Published:** 2023-10-17

**Authors:** Neama Y Hantira, Amal I Khalil, Howaida S Saati, Hend A Ahmed, Fathia K Kassem

**Affiliations:** 1 Community Health Nursing, Alexandria University, Alexandria, EGY; 2 Community Health Nursing, King Saud bin Abdulaziz University for Health Sciences, Jeddah, SAU; 3 Psychiatry and Mental Health Nursing, King Saud bin Abdulaziz University for Health Sciences, Jeddah, SAU; 4 Nursing, Menoufia University, Shibin El Kom, EGY; 5 Oncology Nursing, King Saud bin Abdulaziz University for Health Sciences, Jeddah, SAU; 6 Nursing Education, Damanhour University, Damanhour, EGY; 7 Public Health Nursing, King Abdulaziz University, Jeddah, SAU

**Keywords:** nutritional knowledge, dietary practices, lifestyle habits, food addiction, adolescent nutrition

## Abstract

Background: Changing lifestyles and food habits have an impact on both nutrient requirements and intake among adolescents. The aim of this study is to assess the level of knowledge, habits, practices, and the presence of food addiction among adolescents residing in Damanhur City.

Methods: A descriptive correlational study design is employed to collect data from 363 adolescents selected conveniently from two youth centers in Damanhur, Egypt. Four tools are used: a demographic questionnaire, the Adolescent Food Habits Checklist (AFHC), the General Nutritional Knowledge Assessment Questionnaire (GNKQ), and the Yale Food Addiction Scale version 2.0 (YFAS 2.0).

Results: The age of the participating adolescents ranges from 10 to 19 years. More than half of the participants (51.8%) reported choosing low-fat foods. Additionally, around one-third of the adolescents (34.7%) meet the diagnostic criteria for food addiction. However, there is no statistically significant association found between food addiction and adolescents' eating habits and practices.

Conclusion and recommendations: Most of the studied adolescents exhibit unhealthy eating practices. Food addiction is identified as a significant health concern among this population. Therefore, it is highly recommended to provide nutritional education for adolescents and their families and implement school-based strategies to promote healthy eating habits.

## Introduction

Nutrition is a vital factor in human well-being, and assessing a country's health status involves evaluating its population's nutritional status [1]. Adolescent obesity is a global health concern linked to various adverse health outcomes, including chronic diseases like diabetes and cardiovascular disorders, which affect academic achievement and communities economically [2].

Various heterogeneous factors contribute to the high prevalence of obesity among adolescents, including poor adherence to healthy dietary recommendations and unhealthy personal eating habits, such as the consumption of junk food. One significant factor in the development of obesity is food addiction, which refers to a constant preoccupation with what, when, and how to eat, as well as compulsive overeating behaviors, secretive actions related to food, and an inability to stop or regulate eating [3].

Food addiction involves a psychological and emotional dependence on specific foods and substances, albeit distinct from substance addiction. Consumption of these foods’ triggers pleasure and reward centers in the brain, akin to substances [4]. The concept of food addiction, which has existed for over six decades, suggests highly palatable foods, rich in sugars and fats, can be as addictive as drugs, influencing similar neurocognitive processes [5]. This implies that food addiction may contribute to obesity by affecting the brain's control over food intake, though it remains a subject of debate influenced by complex psychological factors [6].

Food addiction is a growing global concern, notably in the United States, where it has reached epidemic proportions. Despite its substantial impact on well-being, food addiction often goes unrecognized. The Substance Abuse and Mental Health Services Administration (SAMHSA) reports that around 21 million individuals in the United States grapple with various forms of addiction, with one-third of the population directly affected by food addiction's connection to obesity [7].

In Egypt and the Middle East, while the prevalence of adolescent obesity and its associated health consequences have been a concern, there is limited research on the specific topic of food addiction and its impact on eating habits among adolescents. This study aims to bridge this gap in the existing literature by conducting a comprehensive investigation into food habits, practices, and food addiction patterns among adolescents in Damanhur City, Egypt.

Individuals with food addiction develop a reliance on specific foods to experience pleasure, leading to continued consumption even when not hungry. This excessive food intake can result in adverse effects on physical health, emotional well-being, and social interactions, including digestive issues, cardiovascular problems, obesity, diminished self-esteem, depressive symptoms, and social isolation [8]. Despite these negative consequences, individuals with food addiction persist in these behaviors driven by the pursuit of pleasurable sensations.

Improving food knowledge and practices is crucial for modifying eating patterns. Food knowledge shapes positive attitudes toward healthy eating, influencing preferences among adolescents [9]. Food practices encompass aspects like food purchasing, meal patterns, eating habits, and adopting healthy behaviors to meet dietary recommendations. Nutrition literacy, offering information to guide food choices and influence eating practices, plays a pivotal role [10]. Healthy food practices involve reducing the consumption of fats, sugars, and salt in commonly consumed foods, such as instant noodles, processed items, confectioneries, and soft drinks [11]. Additionally, embracing healthier cooking techniques, both at home and in restaurants, is essential. Local health authorities provide valuable recommendations, including the use of low-fat cooking methods like steaming and braising and choosing natural herbs and spices as flavor enhancers for home-cooked meals [12].

Inadequate knowledge about basic food and nutrition information hinders adolescents from making healthy choices in line with recommended guidelines for optimal health. This knowledge gap results in suboptimal diets among adolescents. Community and psychiatric mental health nurses play a vital role in addressing this addiction by understanding adolescents' eating habits and promoting healthy behaviors. They provide relevant health education sessions aimed at effectively transforming unhealthy eating habits and equipping adolescents with the skills to develop lifelong healthy eating habits [2]. To assess food habits, practices, and food addiction patterns among adolescents in Damanhur City, a study was conducted.

Significance of the study

This study on food knowledge, habits, practices, and addiction among adolescents holds significant importance for several reasons. First, adolescence is a critical period for establishing long-term eating behaviors and habits. Poor eating habits formed during this stage can have detrimental effects on health outcomes in later years, including obesity and diet-related diseases [13]. By examining the food knowledge, habits, and practices of adolescents, this study's authors provide valuable insights into their nutritional behaviors during this vulnerable period.

Second, the study's focus is on the prevalence of food addiction among adolescents. Food addiction is a growing concern, and understanding its prevalence and associated factors among this age group is crucial. By shedding light on the extent of food addiction and its impact on adolescents' lives, this study's authors contribute to the existing literature and highlight the need for early detection and intervention [14]. Furthermore, the researchers emphasize the role of the family and school health nurse in promoting healthy eating and active living among adolescents. They recognize the importance of strengthening the support system around adolescents and integrating health education programs that address nutrition and food addiction specifically tailored for this age group [15].

The findings of this study can inform the development of targeted interventions and strategies to improve adolescents' eating behaviors and reduce the incidence of food addiction. By identifying knowledge gaps and areas where improvements are needed, policymakers, healthcare professionals, and educators can implement effective measures to promote healthy eating habits and overall well-being among adolescents [16]. Overall, this study contributes to the understanding of the intersection among eating behaviors, mental health, and addiction among adolescents. Its findings have practical implications for public health initiatives, educational programs, and healthcare interventions aimed at improving the nutritional status and overall health outcomes of adolescents [16].

Aim of the study

The objective of this research is to evaluate the levels of knowledge, food habits and practices, and food addiction patterns among adolescents residing in Damanhur City, Egypt.

Research questions

The study's research questions are as follows: What is the extent of nutritional knowledge among adolescents in Damanhur City? How do adolescents in Damanhur City engage in eating habits and practices? What are the patterns of food addiction observed among adolescents in Damanhur City?

## Materials and methods

Theoretical framework

The social-ecological model (SEM) serves as the theoretical framework for this study, aiming to comprehend food habits, practices, and addiction among adolescents [17]. This model recognizes that individuals' behaviors, including their dietary choices and addictive tendencies, are influenced by multiple levels of the environment. At the individual level, personal factors such as age, gender, genetics, and psychological characteristics play a role in shaping food habits and addiction tendencies during adolescence [17]. The interpersonal level emphasizes the influence of social relationships, including peers, family members, and cultural norms, on dietary choices and food-related behaviors [18]. The community level considers broader social and environmental factors, such as neighborhood characteristics, access to healthy food, and cultural norms, while the societal level focuses on economic, policy, and media influences on food choices [19]. By incorporating the SEM, the researchers recognize the importance of addressing multiple levels of influence to promote healthier food choices and prevent food addiction among adolescents.

**Figure 1:**
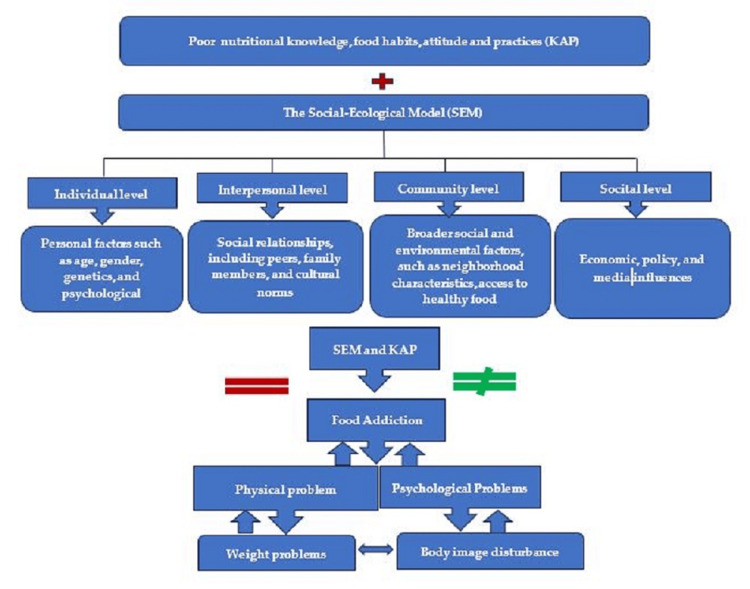
Theoretical framework for adolescent interview: knowledge, food habits and practices, and food addiction model Developed by first author (2023) SEM: social-ecological model, KAP: knowledge, attitude, and practices

The study also incorporates the knowledge, attitude, and practices (KAP) theory, which underscores the impact of knowledge, habits, and practices on adolescent nutrition. This connection is significant because it can contribute to the development of food addiction and negatively affect physical and psychological well-being. Poor physical health increases the risk of noncommunicable diseases, whereas poor psychological health can lead to body image issues. The KAP theory provides a framework for understanding behavior change, emphasizing the acquisition of knowledge, development of attitudes/beliefs, and adoption of new practices/behaviors [18]. Through the examination of knowledge deficiencies and the utilization of the SEM model to identify the habits, practices, and existence of food addiction among adolescents, the results of this study can contribute to the creation of tailored interventions and strategies. These interventions aim to enhance the eating behaviors of adolescents and mitigate the occurrence of food addiction (Figure 1) [19].

Materials

Research Design

A cross-sectional descriptive correlational research design was used to achieve the purpose of this study.

Study Settings

To conduct the current study, two youth centers were selected from Damanhur City with the highest attendance rate of adolescents according to the Sport and Youth Directorate's 2018 data, namely, the Damanhur City Youth Center and the Al Horya Youth Center. Damanhur City was known in the ancient Egyptian language as "the city of the god Horus." It is the capital of Al-Buayrah governorate in the western Nile River delta, Lower Egypt. It is located in the western Delta, about 160 kilometers northwest of Cairo and about 70 miles east of Alexandria. Its name is derived from the ancient Egyptian Timinhor ("City of Horus") and has historically applied to several centers in Egypt, mostly in the delta.

Subjects

From the aforementioned youth centers, 363 teenagers who met the inclusion requirements and were willing to participate, ranging in age from 10 to 19, were selected. This sample size was determined using the Epi Info 7 statistical program (Centers for Disease Control and Prevention, Atlanta, Georgia) with the parameters of a total population of 6,568 adolescents, an expected frequency of 50%, a margin of error of 5%, and a confidence level of 95%, resulting in a minimum sample size of 363 adolescents.

By using the proportional allocation method, the adolescents were selected randomly from each selected youth center based on the following formula. The sample size is distributed as follows (Table 1):

**Table 1:** Distribution of the selected youth centers in Damanhur City

A selected sample of adolescents included in the study	Total number of adolescent attendees	Randomly selected youth center
(4518 × 363) ÷ 6568 = 250	4,518	Damanhur City Youth Center
(2050 × 363) ÷ 6568 = 114	2,050	Al Horya Youth Center
363	6,568	Two centers

Tools of the study

To collect the required data from the study subjects, the following tools were used:

Tool I: Adolescents' sociodemographic characteristics and health profile: a structured interview schedule. It was developed by the researcher after reviewing recent literature to collect the required data. It included the following parts:

Part I: Sociodemographic characteristics: This part consists of sociodemographic data about the adolescent, including age, sex, birth order, religious affiliation, family income, and place of residence.

Part 2: Health profile: This part is concerned with anthropometric data, which were collected based on the standard methods. The BMI formula indicates measuring weight and height and then estimating the BMI as the best method of measuring nutritional status. It was calculated by using the following formula (weight in kilograms divided by height in meters squared). Then the international classification of the BMI category by MOH and El-Zanaty Associate (2015) was used (Table 2) [20].

**Table 2:** The International Classification of the BMI Categories (MOH and El-Zanaty Associate, 2015) [20]

Classification	BMI (Kg/m^2^)
Underweight	<18.5
Normal/average weight	18.5-24.99
Overweight	25.00-29.99
Obese class I	30.00-34.99
Obese class II	35.00-39.99
Obese class III/morbidly obese	≥40

Tool II: Adolescent Food Habits Checklist (AFHC): The AFHC was developed by Johnson, Wardle, and Griffith in 2002 [21]. The checklist was used in the current study to assess the participants' food habits. This tool consists of 23 items that assess an adolescent's healthy eating patterns. Each item represents a specific aspect of healthy eating, and a score of one is given for each healthy response. The total score on the AFHC ranges from 0 to 23. To calculate the median percent score, the total score is divided by 100 and multiplied by the highest possible score of 23. The resulting value represents the median percent score. Based on this median percent score, the total score is categorized into two groups. Adolescents who score below the median percent score are classified as having an unhealthy eating pattern, while those who score equal to or above the median percent score are classified as having a healthy eating pattern.

Tool III: General Nutritional Knowledge Assessment Questionnaire (GNKQ): The GNKQ, developed by Kliemann et al. in 2016 [22] is used to assess the nutritional knowledge of adolescents. The questionnaire consists of four sections: dietary recommendations, food groups, healthy food choices, and diet, diseases, and weight management. The dietary recommendations section includes 18 items, with a total score ranging from 0 to 18 points. The food groups section comprises 36 items, with a total score ranging from 0 to 36 points. The healthy food choices section consists of 13 items, with a total score ranging from 0 to 13 points. Finally, the diet, diseases, and weight management section includes 21 items, with a total score ranging from 0 to 21 points. In each section, a score of 1 is assigned for a correct and complete answer, whereas a score of 0 is given for a wrong answer or if the respondent indicates that they do not know the answer. The total knowledge score on the GNKQ ranges from 0 to 88 points, which is calculated by summing the scores across all sections. To interpret the total score, it is converted into a percentage. A total score below 50% is considered poor knowledge, a score equal to or more than 50% but less than 75% is categorized as fair knowledge, and a score of 75% or more is classified as good knowledge.

Tool IV: Yale Food Addiction Scale version 2.0 (YFAS 2.0): The YFAS 2.0 was developed by Gearhardt et al. (2016) [23] and used to assess symptoms of food addiction based on the Diagnostic and Statistical Manual of Mental Disorders 5th Edition (DSM-5) criteria for substance use disorder. The scale consists of 35 self-report items that evaluate food addiction symptoms experienced over the past year.

Each item is rated on an 8-point scale, ranging from 0 (never) to 7 (every day). Based on the cutoff defined by Gearhardt et al. (2016) [23], a dichotomous rating can be assigned to each item, indicating whether it is endorsed or not. A criterion is considered met if one or more items representing that symptom criterion are endorsed. The YFAS 2.0 offers two scoring options: a symptom counts version and a diagnostic version. In the symptom count version, the total number of criteria met is calculated, along with clinical significance, resulting in a range of 0-11. The diagnostic version considers the diagnosis of food addiction when two or more criteria are met, in addition to clinically significant impairment and distress.

Additionally, YFAS 2.0 allows for the calculation of severity levels of food addiction. Mild severity is defined as meeting two to three symptom criteria plus experiencing impairment or distress. Moderate severity is indicated by meeting four to five symptom criteria plus impairment or distress. Severe severity is identified when six or more symptom criteria are met along with impairment or distress. The YFAS 2.0 is a valuable tool for assessing food addiction symptoms and their severity, providing insights into the presence and impact of food addiction among individuals [23].

Methods

The study followed the following procedures:

Administrative Process

Approval was sought from the Directorate of Youth and Sports in Al-Buayrah Governorate by submitting an official letter from the Faculty of Nursing at Damanhur University. Approval letters were received from the Directorate of Youth and Sports and directed to the directors of the selected youth centers. Meetings were conducted with the directors of the youth centers to explain the study's objectives and seek their cooperation during data collection.

Development of Study Tools

The researchers' tools were translated into Arabic by the researcher. The content validity of the tools was assessed by a panel of five experts in community health nursing from the Faculty of Nursing. Modifications were made to the tools based on the experts' recommendations, including removing unnecessary details and adjusting the phrasing of questions to be suitable for adolescents of all levels. Reliability tests were conducted for Tool II, Tool III, and Tool IV, with Cronbach's alpha values of 0.90, 0.87, and 0.86 respectively.

Pilot Study

A pilot study was conducted on 45 adolescents, representing 10% of the total sample, who were selected from different youth centers and excluded from the main study. The pilot study was aimed at identifying any obstacles or problems in data collection, evaluating the clarity of the questions, and estimating the time required to complete the tools. Based on the pilot study findings, the tools were reviewed, and necessary modifications were made.

Data Collection Procedure

Data collection took place over a period of four months, from September 2019 to December 2019. Adolescents were selected in each setting based on the inclusion criteria. Structured individual interviews were conducted with both male and female adolescents in the selected youth centers, using the study tools. Before each interview, the researchers introduced themselves, explained the purpose of the interview, and assured the participants of the anonymity and confidentiality of their data. The structured interviews lasted approximately 30-40 minutes for each adolescent.

Statistical Analysis

Once the data were collected, they were coded and transformed into a format suitable for computer input. Data entry was followed by a thorough checking and verification process to identify and correct any errors. SPSS Statistics version 23 (IBM Corp. Released 2015. IBM SPSS Statistics for Windows, Version 23.0. Armonk, NY: IBM Corp.) was used for data analysis. Frequency analysis, cross-tabulation, and manual revision were conducted to ensure the accuracy of the entered data. Descriptive statistics included percentages, frequencies, mean, and standard deviation, while inferential statistics included the chi-square test and Fisher's exact test to analyze the variables. The level of significance for this study was set at p<0.05.

Ethical considerations

Prior permission was obtained from the relevant authorities to collect data from the selected settings. Written informed consent was obtained from each participating adolescent after providing a clear explanation of the study's purpose and assuring them that their data would be used solely for research purposes. The directors of the selected settings were informed about the data collection schedule. Participants' privacy, anonymity, and confidentiality of the data were strictly maintained. Participation in the study was voluntary, and adolescents had the right to withdraw from the research at any point. The Faculty of Nursing Damanhur University issued the approval of protocol ID#1342 (IRB:/0942/21).

## Results

Table 3 presents the demographic characteristics of the studied adolescents. The mean age of the participants was 13.8 ± 2.1 years. Approximately 165 (45.5%) of them belonged to the early-age adolescent group, while 164 (45.2%) were classified as middle-aged adolescents. A smaller proportion, around 34 (9.4%), fell into the late-age adolescent category. The vast majority of the adolescents 352 (97%) identified themselves as Muslims. In terms of education, more than two-thirds, 225 (62.0%), had completed their preparatory education. Among the participants, 191 (52.6%) were female. Around one-third of the adolescents were either the first or second child in their families, accounting for 121 (33.3%) and 118 (32.5%), respectively. Concerning family income, less than two-thirds, 225 (62%), of the adolescents reported not having any income. The majority of the studied adolescents, 344 (94.8%), resided in urban areas. Only a small proportion, 32 (8.8%), had attended nutritional courses, whereas the remaining 331 (91.2%) had not.

**Table 3:** Distribution of adolescents and families according to their sociodemographic characteristics

Adolescent’s characteristics	Total n=363	
	No.	%
Age in years		
Early adolescent (10-13)	165	45.5
Middle adolescent (14-16)	164	45.2
Late adolescent (17-19)	34	9.4
Mean ± SD	13.8 ± 2.1	
Religion		
Muslim	352	97
Christian	11	3
Gender		
Female	191	52.6
Male	172	47.4
Educational level		
Read and write	22	6.1
Primary	62	17.1
Preparatory	225	62
Secondary	54	14.9
Birth order		
The first	121	33.3
The second	118	32.5
The third	78	21.5
Other	46	12.7
Family income		
Enough	50	13.8
Not enough	225	62
Enough and safe	88	24.2
Place of residence		
Urban	344	94.8
Rural	19	5.2
Attendance of nutritional courses		
No	331	91.2
Yes	32	8.8

Table 4 provides information on the BMI and weight perception of the adolescents, as well as their health history. Regarding BMI, the majority of adolescents, 184 (50.7%), were classified as normal, with a BMI ranging from 18.5 to 24 kg/m2. Around 101 (27.8%) were categorized as underweight (BMI less than 18.5 kg/m2), while only 47 (12.9%) were classified as overweight (BMI between 18.5 and 24.9 kg/m2). A small percentage of adolescents fell into the obese categories, with 17 (4.7%) in class I (BMI between 30 and 34.99 kg/m2), 4 (1.1%) in class II (BMI between 35 and 39.99 kg/m2), and 10 (2.8%) in class III (BMI equal to or greater than 40 kg/m2).

**Table 4:** Distribution of adolescents according to their health profile, health history, and practicing of exercises #: multiple responses

Items	Total n=363	
	No.	%
BMI (kg/m^2^)		
Underweight (<18.5)	101	27.8
Normal (18.5-24.99)	184	50.7
Overweight (25.00-29.99)	47	12.9
Obese class I (30.00-34.99)	17	4.7
Obese class II (35.00-39.99)	4	1.1
Obese class III (≥40)	10	2.8
Evaluation of Weight from Adolescents' Perspective		
Less than normal/underweight	66	18.2
Your weight is normal/normal weight within the average	213	58.7
Over the normal/above average	52	14.3
Obesity	32	8.8
Previous health problems		
Yes	105	28.9
No	258	71
Types of health problems#	n=105	
Anemia	93	88.5
Diabetes	30	28.5
Thyroid disorders	28	26.6
Cholecystitis	24	22.8

Regarding weight perception, more than half of the adolescents, 213 (58.7%), perceived their weight to be normal or within the average range. A smaller proportion, 66 (18.2%), considered themselves to be underweight or less than normal, while 52 (14.3%) believed their weight was above the average. Only 32 (8.8%) of the adolescents perceived themselves as having obesity. In terms of health history, the table revealed that anemia was the most commonly reported health problem among adolescents, with a prevalence of 93 (88.5%). Additionally, more than one-quarter, 30 (28.5%), of them reported suffering from diabetes, 28 (26.6%) had thyroid disorders, and less than one-quarter, 24 (22.8%), had experienced cholecystitis in the past.

According to Table 5, approximately half, 173 (47.7%), of the adolescents had inadequate knowledge regarding dietary recommendations. Moreover, more than two-thirds, 242 (66.7%), demonstrated poor knowledge concerning food groups, whereas 146 (40.2%) of the adolescents exhibited good knowledge about healthy food choices. However, more than two-thirds, 236 (65%), of them had insufficient knowledge regarding diet, diseases, and weight management. When considering the overall knowledge scores, it was found that less than two-thirds, 221 (60.8%), of the adolescents had inadequate knowledge, one-third, 120 (33.1%), had moderate or fair knowledge, and only a small percentage, 22 (6.1%), had good knowledge.

**Table 5:** Distribution of adolescents according to their general nutritional knowledge

Nutritional category	Level of adolescent knowledge					
	Poor		Fair		Good	
	No.	%	No.	%	No.	%
Dietary recommendations	173	47.7	110	30.3	80	22.0
Food groups	242	66.7	79	21.8	42	11.6
Healthy food choices	99	27.3	118	32.5	146	40.2
Diet, diseases, and weight management	236	65	114	31.4	13	3.5
Total knowledge score	221	60.8	120	33.1	22	6.1

Table 6 shows the distribution of the studied adolescents regarding the food habits category; more than half, 217 (59.8%), of them followed unhealthy eating practices, while about two-fifths, 146 (40.2%), of them followed healthy eating practices.

**Table 6:** Distribution of adolescents according to their food habits and practices category

Food habits and practices category	Total n=363	
	No.	%
Unhealthy eating habits	217	59.8
Healthy eating habits	146	40.2

Table 7 presents the distribution of the studied adolescents based on food addiction. Approximately two-thirds, 237 (65.2%), of the adolescents did not exhibit any food addiction. However, more than one-tenth of them had a mild food addiction, 57 (15.7%), or a moderate food addiction, 44 (12.1%). Only 25 (7%) of the adolescents were classified as having a severe food addiction.

**Table 7:** Distribution of adolescents according to their degree of food addiction

Degree of food addiction	Total n=363	
	No.	%
No food addiction	237	65.2
Mild food addiction	57	15.7
Moderate food addiction	44	12.1
Sever food addiction	25	7

Table 8 presents the relationship between the degree of food addiction in adolescents and their socio-demographic characteristics. The table indicates that there is no statistically significant association between the adolescents' demographic characteristics and their degree of food addiction. The data shows that there is no significant difference in the prevalence of food addiction between adolescents who had attended nutritional courses, 27 (79.4%), and those who had not attended nutritional courses, 210 (63.8%). The association between attending nutritional courses and the degree of food addiction among adolescents was not found to be statistically significant (X2=7.298, P=0.063).

**Table 8:** Association between the degree of food addiction among the adolescents and their basic characteristics X^2^: chi-square test, *: statistically significant at p≤0.05

Adolescent’s characteristics	Degree of food addiction								Total N=363	Test of significance
	No addiction		Mild		Moderate		Sever			
	No.	%	No.	%	No.	%	No.	%	No.	
Age in years										
Early adolescent (10-13)	115	69.7	22	13.3	14	8.5	14	8.5	165	X^2^=6.707 P=0.349
Middle adolescent (14-16)	100	61.0	30	18.3	25	15.2	9	5.5	164	
Late adolescent (17-19)	22	64.7	5	14.7	5	14.7	2	5.9	34	
Religion										
Muslim	231	65.6	55	15.6	43	12.2	23	6.5	352	X^2=^2.432 P=0.488
Christian	6	54.5	2	18.2	1	9.1	2	18.2	11	
Gender										
Female	129	67.5	28	14.7	21	11.0	13	6.8	191	X^2^=1.018 P=0.797
Male	108	62.8	29	16.9	23	13.4	12	7.0	172	
Educational level										
Read and write	17	77.3	2	9.1	2	9.1	1	4.5	22	X^2^=10.601 P=0.304
Primary	41	66.1	9	14.5	5	8.1	7	11.3	62	
Preparatory	143	63.6	37	16.4	34	15.1	11	4.9	225	
Secondary	36	66.7	9	16.7	3	5.6	6	11.1	54	
Birth order										
The first	75	62.0	20	16.5	16	13.2	10	8.3	121	X^2^=5.439 P=0.795
The second	84	71.2	17	14.4	9	7.6	8	6.8	118	
The third	50	64.1	12	15.4	11	14.1	5	6.4	78	
The fourth	28	60.9	8	17.4	8	17.4	2	4.3	46	
Family income										
Enough	149	66.2	36	16.0	22	9.8	18	8.0	225	X^2^=4.624 P=0.593
Not enough	31	62.0	9	18.0	7	14.0	3	6.0	50	
Enough and safe	57	64.8	12	13.6	15	17.0	4	4.5	88	
Place of residence										
Urban	223	64.8	53	15.4	43	12.5	25	7.3	344	X^2^=2.741 P=0.433
Rural	14	73.7	4	21.1	1	5.3	0	0	19	
Attendance of nutritional courses										
No	210	63.8	57	17.3	39	11.9	23	7.0	329	X^2^=7.298 P=0.063
Yes	27	79.4	0	0	5	14.7	2	5.9	34	

Table 9 presents the relationship among the BMI of adolescents, their health history, and the degree of food addiction. The data indicates that there is no statistically significant association between the adolescents' BMI and their health history concerning the degree of food addiction.

**Table 9:** Association between the degree of food addiction among the adolescents and BMI and health profile X^2^: chi-square test, *: statistically significant at p≤0.05

	Degree of food addiction								Total N=363	Test of significance
	No addiction		Mild		Moderate		Severe			
	No.	%	No.	%	No.	%	No.	%	No.	
BMI (kg/m^2^)										
Underweight (<18.5)	64	63.4	18	17.8	14	13.9	5	5.0	101	X^2^=17.941 P=0.266
Normal (18.5-24.99)	124	67.4	26	14.1	17	9.2	17	9.2	184	
Overweight (25.00-29.99)	28	59.6	11	23.4	6	12.8	2	4.3	47	
Obese class I (30.00-34.99)	10	58.8	2	11.8	4	23.5	1	5.9	17	
Obese class II (35.00-39.99)	2	50.0	0	0	2	50.0	0	0	4	
Obese class III (≥40)	9	90.0	0	0	1	10.0	0	0	10	
Previous health problems										
No	173	67.1	38	14.7	29	11.2	18	7.0	258	X^2^=1.546 P=0.672
Yes	64	61.0	19	18.1	15	14.3	7	6.7	105	
Types of health problems										
Anemia										
No	180	66.7	42	15.6	30	11.1	18	6.7	270	X^2^=1.282 P=0.733
Yes	57	61.3	15	16.1	14	15.1	7	7.5	93	
Diabetes										
No	219	65.8	53	15.9	40	12.0	21	6.3	333	X^2^=2.270 P=0.518
Yes	18	60.0	4	13.3	4	13.3	4	13.3	30	
Thyroid disorders										
No	221	66.0	49	14.6	42	12.5	23	6.9	335	X^2^=4.132 P=0.248
Yes	16	57.1	8	28.6	2	7.1	2	7.1	28	
Cholecystitis										
No	223	65.8	50	14.7	42	12.1	24	7.1	339	X^2^=3.738 P=0.291
Yes	14	58.3	7	25.0	2	8.3	1	4.2	24	

Table 10 demonstrates a statistically significant association between the adolescents' food knowledge and their degree of food addiction (X2=16.375, P=0.012). However, no significant association was found between the adolescents' food habits and their degree of food addiction.

**Table 10:** Association between the degree of food addiction among adolescents and their general nutritional knowledge levels and food habits/practices X^2^: chi-square test, *: statistically significant at p≤0.05

Items	Degree of food addiction								Total n=363	Test of significance
	No addiction		Mild		Moderate		Sever			
	No.	%	No.	%	No.	%	No.	%	No.	
Adolescents' general nutritional knowledge levels										
Poor	104	61.5	30	17.8	29	17.2	6	3.5	169	X^2^=16.375 P=0.012*
Fair	117	68.8	24	14.1	11	6.5	18	10.6	170	
Good	16	66.7	3	12.5	4	16.7	1	4.1	24	
Adolescents' food habits/practices										
Unhealthy eating	103	70.5	21	14.4	15	10.3	7	4.8	146	X^2^=3.545 P=0.315
Healthy eating	134	61.8	36	16.6	29	13.4	18	8.3	217	

Table 11 provides the following findings: There is a statistically significant association between the age of adolescents and their level of knowledge. Among late adolescents aged 17-19 years, 21 (61.8%) had a poor level of knowledge, while among early adolescents aged 10-13 years, the percentage was 65 (39.4%) (X2=10.766, P=0.029). There is no statistically significant association between the religion of adolescents and their level of knowledge. Among Muslim adolescents, 164 (46.6%) had a poor level of knowledge compared to 5 (45.5%) among Christian adolescents (X2=0.113, P=0.945). In addition, there is no statistically significant association between the gender of adolescents and their level of knowledge. A higher percentage of females, 98 (51.3%), had a poor level of knowledge compared to males, 71 (41.3%) (X2=3.706, P=0.157).

**Table 11:** Association between the socio-demographic characteristics of adolescents and their knowledge level X^2^: chi-square test, *: statistically significant at p≤0.05

Sociodemographic characteristics	Knowledge level						Total n=363	Test of significance
	Poor knowledge		Fair knowledge		Good knowledge			
Age in years	No.	%	No.	%	No.	%	No.	
Early adolescent (10-13)	65	39.4	84	50.9	16	9.7	165	X^2^=10.766 P=0.029*
Middle adolescent (14-16)	83	50.6	73	44.5	8	4.9	164	
Late adolescent (17-19)	21	61.8	13	38.2	0	0	34	
Religion								
Muslim	164	46.6	165	46.9	23	6.5	352	X^2^=0.113 P=0.945
Christian	5	45.5	5	45.5	1	9.1	11	
Gender								
Female	98	51.3	81	42.4	12	6.3	191	X^2^=3.706 P=0.157
Male	71	41.3	89	51.7	12	7.0	172	
Educational level								
Read and write	10	45.5	8	36.4	4	18.2	22	X^2^=19.565 P=0.003*
Primary	114	50.7	42	67.7	1	1.6	62	
Preparatory	26	48.1	94	41.8	17	7.6	225	
Secondary	19	30.6	26	48.1	2	3.7	54	
Birth order								
The first	54	44.6	59	48.8	8	6.6	121	X^2^=4.992 P=0.545
The second	56	47.5	52	44.1	10	8.5	118	
The third	42	53.8	33	42.3	3	3.8	78	
The fourth	17	37.0	26	56.5	3	6.5	46	
Family income								
Enough	103	45.8	107	47.6	15	6.7	225	X^2^=1.273 P=0.866
Not enough	23	46.0	25	50.0	2	4.0	50	
Enough and safe	43	48.9	38	43.2	7	8.0	88	
Place of residence								
Urban	160	46.5	161	46.8	23	6.7	344	X^2^=0.059 P=0.971
Rural	9	47.4	9	47.4	1	5.3	19	
Attendance of nutritional courses								
No	153	46.5	153	46.5	23	7.0	329	X^2^=0.847 P=0.655
Yes	16	47.1	17	50.0	1	2.9	34	
Adolescents' food habits/practices								
Unhealthy eating	71	48.6	63	43.2	12	8.2	146	X^2^=1.887 P=0.389
Healthy eating	98	45.2	107	49.3	12	5.5	217	

That said, there is a statistically significant association between the level of education of adolescents and their level of knowledge. Among adolescents in primary education, 114 (50.7%) had a poor level of knowledge, while among those in secondary education, the percentage was 19 (30.6%) (X2=19.565, P=0.003). Additionally, there is no significant statistical association among the birth order of adolescents, the sufficiency of income, the place of residence, attendance of nutritional courses, and the level of knowledge of adolescents. There is no significant statistical association between the food habits/practices of adolescents and their level of knowledge (X2=1.887, P=0.389).

## Discussion

In recent years, there has been a growing interest in exploring the connection between eating behaviors and mental health, particularly during adolescence, a critical period for establishing nutrition habits [24]. Poor eating habits during this phase can contribute to obesity and diet-related diseases later in life. Additionally, the prevalence of dieting behaviors among adolescents can lead to nutritional deficiencies and eating disorders [25]. Thus, this study aimed to assess the knowledge, food habits, food addiction patterns, and practices among adolescents in Damanhur City. One possible explanation for this apparent gap between knowledge and practice could be the influence of external factors. Adolescents are often exposed to a multitude of environmental influences, including peer pressure, advertising, and the availability of convenience foods. These external factors can significantly impact their food choices and may contribute to unhealthy eating practices, even when they possess knowledge to the contrary.

The study found that over half of the adolescents exhibited unhealthy eating practices, consistent with research conducted in London [26]. This may result from a lack of effective nutritional education programs for adolescents implemented by health authorities. Consequently, adolescents tend to consume excessive fast and processed foods, as well as sugar. Despite their awareness of the health benefits of specific nutrients, they consume insufficient amounts of fruits and vegetables, a persistent issue in developing countries. Furthermore, the concept of food addiction has gained attention, suggesting that highly processed and palatable foods may be addictive and lead to overeating. The study revealed that more than a third of adolescents met the criteria for food addiction according to the Yale Food Addiction Scale (YFAS). Common symptoms included giving up important activities, withdrawal, engaging in risky situations, cravings, excessive time spent on food-related activities, failure to meet obligations, overconsumption, unsuccessful attempts to quit, and tolerance. These findings are consistent with other studies, highlighting the prevalence of food addiction among various populations [14,15]. Addressing food addiction among adolescents is crucial due to its potential consequences. Unhealthy eating habits formed during adolescence can lead to obesity and diet-related diseases, while being underweight can impact energy levels, growth, and nutrient deficiencies. The distribution of weight categories among adolescents in the study aligns with previous research [27]. Furthermore, it is important to note that food addiction and obesity are not always linked; they can occur independently.

These findings underscore the importance of addressing food addiction among adolescents and implementing appropriate interventions. Promoting healthy, balanced diets during adolescence is crucial for maintaining a healthy weight and preventing obesity-related health issues such as diabetes, high blood pressure, and heart disease. Additionally, addressing underweight issues and ensuring adequate nutrient intake is essential for proper growth and development.

The study identified a nonlinear relationship between addictive-like eating and BMI, consistent with previous research [28,29]. Dietary education practices play a vital role in increasing adolescents' awareness of healthy eating behaviors and the impact of food addiction on their health. The study categorized food addiction into degrees, revealing that more than half of the participants did not exhibit food addiction, while some had mild or moderate addiction, and a small percentage suffered from severe food addiction [30].

In summary, the results of this study resonate with the SEM by highlighting the multi-level influences on adolescents' eating behaviors, from individual characteristics to societal factors. Additionally, the findings align with the KAP theory by emphasizing the potential gap between knowledge and dietary practices. Recognizing these frameworks provides a valuable lens through which to interpret and contextualize the study's findings and informs future interventions aimed at improving adolescents' nutritional behaviors.

Moreover, this study provides valuable insights into eating behaviors and food addiction among adolescents aged 10-19 years in selected youth centers in Damanhur. Community health nurses play a pivotal role in understanding adolescents' needs, educating them and their parents about nutritional requirements and the importance of a healthy lifestyle, and providing appropriate dietary recommendations to prevent food addiction among adolescents.

Recommendations

Based on the findings of the study, several recommendations are proposed to address the issues identified among adolescents in Damanhur City. Firstly, it is essential to focus on parental and adolescent education to enhance health promotion activities and meet nutritional needs. Parents should attend nutrition-related courses to improve their understanding of healthy meals and play a significant role in implementing this knowledge when preparing meals for adolescents. Additionally, school-based interventions promoting healthy lifestyles during adolescence should be implemented, with school health nurses developing plans to support students in adopting healthy practices. Schools can provide nutritious breakfast options and organize classes for mothers to enhance dietary knowledge. Mass media should play a role in raising health awareness, and positive role models should be showcased to promote healthy behaviors. Leveraging new technologies like the Internet and social media can effectively disseminate healthy nutrition messages. Routine screening for food addiction in adolescents is recommended, facilitating early detection and tailored nutritional plans. Integrating up-to-date nutrition knowledge into the curriculum and incorporating healthy living practices within the education system is vital. School-based strategies and physical fitness programs should be implemented, and collaboration with non-governmental organizations focused on adolescent health and welfare is crucial for addressing unhealthy eating habits among adolescents. These measures collectively aim to improve the overall health and well-being of adolescents in the community.

Further research suggestions

Based on the outcomes of this study, future research could be focused on three primary areas:

Longitudinal Studies

Conducting long-term, longitudinal studies to track the development and effects of food addiction among adolescents as they transition into adulthood. This research could provide valuable insights into the trajectory of food addiction and its consequences on both physical and mental health over time.

Intervention Studies

Designing and implementing interventions specifically tailored to address food addiction in adolescents. Evaluating the effectiveness of these interventions, such as educational programs, counseling, and behavioral therapies, in reducing food addiction and promoting healthier eating behaviors.

Sociocultural Factors

Investigating the influence of sociocultural factors on the development and maintenance of food addiction among adolescents. This includes exploring the impact of family dynamics, peer influence, media exposure, and socioeconomic status on food addiction. Understanding these contextual factors can guide the development of targeted interventions.

These research areas would collectively contribute to a deeper understanding of food addiction in adolescents and the development of evidence-based strategies for prevention and intervention.

## Conclusions

The study unveiled a significant prevalence of unhealthy eating behaviors and limited nutritional knowledge among surveyed adolescents. These findings underscore the pressing need for nutritional education programs aimed at benefiting adolescents and their families. Additionally, the study's results indicate the presence of food addiction among the adolescents at the Damanhour youth centers. This addiction manifests through various symptoms, with most adolescents experiencing mild food addiction, followed by moderate and severe levels. These findings emphasize the importance of dietary interventions to prevent obesity and chronic illnesses in this age group.
